# Biomarkers for Pulmonary Vascular Remodeling in Systemic Sclerosis: A Pathophysiological Approach

**DOI:** 10.3389/fphys.2018.00587

**Published:** 2018-06-19

**Authors:** Balazs Odler, Vasile Foris, Anna Gungl, Veronika Müller, Paul M. Hassoun, Grazyna Kwapiszewska, Horst Olschewski, Gabor Kovacs

**Affiliations:** ^1^Ludwig Boltzmann Institute for Lung Vascular Research, Graz, Austria; ^2^Division of Pulmonology, Department of Internal Medicine, Medical University of Graz, Graz, Austria; ^3^Physiology, Otto Loewi Research Center, Medical University of Graz, Graz, Austria; ^4^Department of Pulmonology, Semmelweis University, Budapest, Hungary; ^5^Division of Pulmonary & Critical Care Medicine, Johns Hopkins University, Baltimore, MD, United States

**Keywords:** systemic sclerosis, autoimmune, vascular, fibrosis, pulmonary hypertension, PAH, biomarker

## Abstract

Pulmonary arterial hypertension (PAH) is a severe complication of systemic sclerosis (SSc) associated with high morbidity and mortality. There are several biomarkers of SSc-PAH, reflecting endothelial physiology, inflammation, immune activation, extracellular matrix, metabolic changes, or cardiac involvement. Biomarkers associated with diagnosis, disease severity and progression have been identified, however, very few have been tested in a prospective setting. Some antinuclear antibodies such as nucleosome antibodies (NUC), anti-centromere antibodies (CENP-A/B) and anti-U3-ribonucleoprotein (anti-U3-RNP) are associated with PAH while anti-U1-ribonucleoprotein (anti-U1-RNP) is associated with a reduced PAH risk. Anti-endothelin receptor and angiotensin-1 receptor antibodies might be good markers of SSc-PAH and progression of pulmonary vasculopathy. Regarding the markers reflecting immune activation and inflammation, there are many inconsistent results. CXCL-4 was associated with SSc progression including PAH and lung fibrosis. Growth differentiation factor (GDF)-15 was associated with PAH and mortality but is not specific for SSc. Among the metabolites, kynurenine was identified as diagnostic marker for PAH, however, its pathologic role in the disease is unclear. Endostatin, an angiostatic factor, was associated with heart failure and poor prognosis. Established heart related markers, such as N-terminal fragment of A-type natriuretic peptide/brain natriuretic peptide (NT-proANP, NT-proBNP) or troponin I/T are elevated in SSc-PAH but are not specific for the right ventricle and may be increased to the same extent in left heart disease. Taken together, there is no universal specific biomarker for SSc-PAH, however, there is a pattern of markers that is strongly associated with a risk of vascular complications in SSc patients. Further comprehensive, multicenter and prospective studies are warranted to develop reliable algorithms for detection and prognosis of SSc-PAH.

## Introduction

Systemic sclerosis (SSc) is an autoimmune, multiorgan disease characterized by autoimmunity, fibrosis and vascular damage of the skin and other organs, including the lungs. Clinically, SSc is a heterogeneous disease which is classified in two major subtypes based on the extent of skin involvement: limited cutaneous (lcSSc) and diffuse cutaneous SSc (dcSSc) (Leroy et al., 1988). DcSSc patients are characterized by a generalized skin involvement with sometimes rapidly progressive and often fatal organ involvement, while lcSSc patients generally show a slower progression with isolated cutaneous involvement. A third phenotype called systemic sclerosis sine scleroderma (ssSSc) can also be differentiated, if the patients have any of the characteristics features of internal organ involvement without skin involvement. In addition, SSc may overlap with other rheumatic or autoimmune disorders such as rheumatoid arthritis (RA), dermatomyositis or systemic lupus erythematosus (SLE). To make a definitive classification, the criteria of the American College of Rheumatology/European League against Rheumatism (ACR/EULAR) should be applied (van den Hoogen et al., 2013; Jordan et al., 2015).

Pulmonary arterial hypertension (PAH) is a devastating disease which develops on the basis of proliferative vasculopathy of small and medium-sized pulmonary arteries, leading to an increase in mean pulmonary artery pressure (mPAP ≥25 mmHg) at rest. The diagnosis must be confirmed by right heart catheterization (RHC), which besides measuring pulmonary arterial pressure (PAP) allows the determination of pulmonary arterial wedge pressure (PAWP), pulmonary vascular resistance (PVR), and cardiac output (Galiè et al., 2015). Based on the values of the PAWP a pre- (PAWP ≤ 15 mmHg) and post-capillary (PAWP ≥15 mmHg) pulmonary hypertension can be distinguished (Galiè et al., 2015). The life-time prevalence of PAH in SSc patients ranges from 5 to 12% (Avouac et al., 2010; Hao et al., 2015; Kovacs et al., 2017; Morrisroe et al., 2017), while the prognosis of these patients is very poor with about 50% 3-year mortality after PAH diagnosis (Chaisson and Hassoun, 2013; Chung et al., 2014b). SSc-PAH patients have a worse outcome compared to idiopathic (IPAH) or PAH associated with other collagen vascular diseases, such as mixed connective tissue disease (MCTD) or SLE and PAH represents one of the leading causes of death in SSc (Chung et al., 2010, 2014b; Tyndall et al., 2010; Sobanski et al., 2016). Despite the extended involvement of internal organs in dcSSc, PAH occurs more commonly in patients with lcSSc (Denton and Khanna, 2017). In the setting of SSc, primary pulmonary vasculopathy is not the unique cause of pulmonary hypertension (PH). Significant lung disease, which might lead to PH due to hypoxaemia was identified in up to 30–75% of SSc patients complicated by elevated pulmonary arterial pressure (group 3 of the World Classification of PH) (Kowal-Bielecka et al., 2010), although a clear delineation from PAH (group 1) is sometimes difficult to establish. In addition, left ventricular systolic or diastolic dysfunction, which is frequently found in SSc patients, may cause postcapillary PH (group 2). Finally, pulmonary venoocclusive disease, a rare variant of PAH, may be also associated with SSc (Dorfmüller et al., 2007).

There are some differences in the pathogenesis of SSc-PAH as compared to IPAH. As an example, the expression of bone morphogenetic protein receptor 2 (BMPR2) is highly associated with heritable PAH and is often present in IPAH (Rubin, 2017), but this mutation has not been found in SSc-PAH patients, at least in two small genetic studies (Morse et al., 2002).

**Figure 1:**
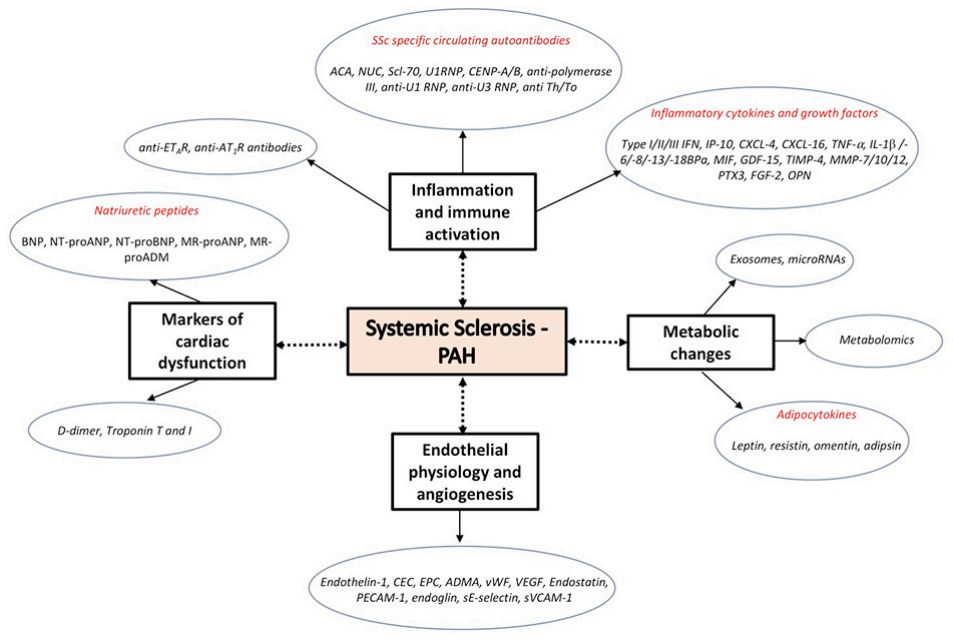
Molecular changes and associated biomarker candidates in the development of pulmonary vascular remodeling.

Recent studies provided novel insight into the key signaling pathways of PAH including the role of endothelial dysfunction, growth factors, inflammation, immune activation, metabolic changes, extracellular remodeling, and the development of heart failure (Figure 1). Considering the fact that PAH is a life-threatening complication of SSc, blood biomarkers of pulmonary vascular involvement, either alone, or in combination with other prognostic clinical parameters may be important tools contributing to earlier diagnosis and targeted treatment. In this review we summarize blood biomarkers associated with key changes reflecting the molecular pathology of pulmonary vascular abnormalities in SSc.

## Inflammation and immune activation

There is an increasing body of evidence for an inflammatory component in the patomechanism of pulmonary hypertension. The presence and exubarence of inflammatory cells and their interactive interplay may provide a missing link between PAH, autoimmunity, and inflammation (Marsh et al., 2018). However, the detailed and comprehensive description of the interaction between the inflammatory cells and proinflammatory processes is still difficult.

The imbalance and dysregulation of immune function and tolerance may lead to autoimmunity and chronic inflammation involving different types of immune cells and chemokines. Autoimmunity and immune activation of both the innate and adaptive immune system may play a role in the early development of SSc. The subsequent activation of immune cells and fibroblasts may contribute to the pathogenesis of SSc and accelerated fibrogenesis and extracellular matrix deposition (Varga and Abraham, 2007). Immunological and inflammatory aspects of the disease may be therefore correlated with vascular and fibrotic manifestations and reflected by changes in the levels of corresponding circulating biomarkers (Gu et al., 2008; Denton, 2015), although most of these markers are not specific and often not based on robust studies.

### SSc specific circulating autoantibodies

As recently added to the ECR/EULAR criteria, the presence of highly SSc-specific circulating autoantibodies such as anti-topoisomerase 1 (ATA), anti-centromere (CENP, ACA), and anti-RNA polymerase III are used for the diagnosis of the disease (van den Hoogen et al., 2013). Specific autoantibodies indicating pulmonary vascular involvement, pulmonary disease progression or treatment response have not been included in this statement because they have not been confirmed in prospective studies. Over 85% of SSc patients present with circulating antibodies and there is emerging evidence that these antibodies are present in early stages of the disease highlighting their role as both pathogenetically important factors and early diagnostic biomarkers (Choi and Fritzler, 2016).

Over 90% of SSc patients present with antinuclear antibodies (ANAs), however, there is no association with the development of PAH (Sweiss et al., 2010). According to data from the Pulmonary Hypertension Assessment and Recognition of Outcomes in Scleroderma (PHAROS) Registry, the prevalence of anticentromere (ACA) and nucleolar antibodies (NUC), in SSc-PAH as compared to SSc without PAH, was elevated (35–37 and 24%, respectively) but not the prevalence of Scl-70 (7%) and U1RNP (5%) antibodies. No association was found between any of these biomarkers and survival (Chung et al., 2014a; Hinchliff et al., 2015). Accordingly, patients having ACA, CENP-A and/or CENP-B were more likely to have PAH but less likely to have ILD (Hudson et al., 2012).

There have been conflicting results regarding anti-polymerase III and PAH. A large longitudinal study showed that the presence of anti-polymerase III is a positive predictor for PAH (Nihtyanova et al., 2014). However, this could not be confirmed in another prospective patient cohort (Hoffmann-Vold et al., 2017). A study investigating 342 CTD-associated PAH patients found that Anti-U1 RNP positivity was associated with decreased mortality in CTD-associated PAH patients, even after correction for hemodynamic impairment (Sobanski et al., 2016). In contrast, SSc patients with anti-U3 RNP positivity were more frequently affected by PAH which was the most common cause of death in this patient group (Okano et al., 1992; Aggarwal et al., 2009). A study comparing lcSSc patients with anti-Th/To-positivity and CENP-positivity found that both groups presented with a high frequency of PAH, while the frequency of ILD was higher in the anti-Th/To group (Mitri et al., 2003). Among the larger PAH screening algorithms, only the DETECT (Evidence-Based Detection of Pulmonary Arterial Hypertension in Systemic Sclerosis) algorithm included an autoantibody (ACA) as criteria for identification of PAH in SSc (Hao et al., 2015). Autoantibody positivity and their association with survival in SSc-ILD and SSc-PAH patients are listed in Table 1.

**Table 1:** Autoantibody positivity and association with survival in SSc-ILD and SSc-PAH patients.

**Reference**	**Antibody**	**Prevalence of PAH in patients with antibody positivity (No. of patients)**	**Prevalence of ILD in patients with antibody positivity (No. of patients)**	**Association with survival**	**Independent predictive ability for PAH (Odds ratio)**
Hinchliff et al., 2015^*^	ACA	37% (162)	–	No	–
	ANA	24%	–	No	–
	Scl-70	7%	–	No	–
	U1RNP	5%	–	No	–
	RNA pol III	6%	–	No	–
Sobanski et al., 2016^#^	Anti-U1 RNP	11% (342)	–	No (*p* = 0.055)	
Okano et al., 1992	Anti-U3 RNP	17% (24)	25% (24)	–	–
Aggarwal et al., 2009	Anti-U3 RNP	31% (86)	36% (97)	–	–
Mitri et al., 2003	Anti Th/To	28% (87)	48% (87)	–	–
	ACA	19% (306)	13% (306)	–	–
Becker et al., 2014	Anti-ET_A_R	–	–	Yes	2.7
	Anti-AT_1_R	–	–	Yes	1.053

*ACA, anticentromere antibody; anti-ETAR, endothelin-1 type A receptor; AT1R, angiotensin II type 1 receptor; ANA, antinucleolar antibody; ILD, interstitial lung disease; PAH, pulmonary arterial hypertension; RNA pol III, RNA polymerase III; Scl-70, antitopoisomerase antibody*.

* *SSc vs. SSc-PAH*.

# *CTD-PAH patients*.

### Endothelin-1 type a receptor and angiotensin receptor type-1 antibodies

Autoantibodies against endothelin receptor type A (anti-ET_A_R Ab) and angiotensin receptor type-1 (anti-AT_1_R Ab) may affect inflammation and fibrotic processes by direct receptor activation thus causing vasoconstriction and proliferation (Kill et al., 2014; Cabral-Marques and Riemekasten, 2016). These autoantibodies are more frequent in patients with CTD-PAH compared to other forms of PH and might be diagnostic and prognostic biomarkers in SSc-PAH and/or CTD-PAH (Becker et al., 2014). In addition, it has been shown that anti-ET_A_R Ab may identify patients at risk for the development of subsequent digital ulceration. Furthermore, SSc patients with loss of capillaries showed a strong association between the presence of an antibody titer and digital ulcerations or PH (Avouac et al., 2015b).

### Interferons

A member of the interferon family, the type I interferon (type I IFN) has a central role in the innate immune response to viral infections, while type I IFN therapy may represent a risk factor for PAH (Galiè et al., 2015). In a recent study, investigating the role of type I IFN in PAH, serum levels of type I, II, and III IFN were found to be increased in patients with SSc-PAH (George et al., 2014). In addition, the serum interferon γ inducible protein 10 (IP10/CXCL10) was positively correlated with hemodynamic parameters, 6 minute walking distance test (6MWT), brain natriuretic peptide (BNP) and cardiac index (CI) (George et al., 2014). In another study there was also an association between IP-10 and PAH in SSc patients (Eloranta et al., 2010).

### Chemokines

Chemokines belong to a protein family with a major role in leukocyte activation and chemoattraction, but they may also play an important role in angiogenesis (Koch et al., 1992; Strieter et al., 1995). CXCL4 is a chemokine with potent antiangiogenic properties which is secreted by megakaryocytes and plasmocytoid dendritic cells. The serum level of CXCL4 was markedly elevated in SSc patients and associated with PAH and lung fibrosis development (van Bon et al., 2014). The proangiogenic receptor CXCR6 ligand CXCL16 was also elevated in patients with SSc-PAH, however, correlation analysis with hemodynamic parameters was not performed (Rabquer et al., 2011).

### Inflammatory cytokines and growth factors

In SSc-PAH patients, a large number of pro-inflammatory cytokines have been recorded to be increased. The tumor necrosis factor alpha (TNF-α), interleukin (IL)-1β, IL-6, IL-8, and IL-13 are elevated in the serum and plasma of lcSSc-PAH patients (Pendergrass et al., 2010; Christmann et al., 2011). IL-6 is increased in MCTD-PAH patients compared to those without PAH (Nishimaki et al., 1999). The IL-18-binding isoform a (IL-18BPa) is elevated in SSc patients compared to healthy controls and positively correlated with SPAP and mPAP (Nakamura et al., 2016a). The macrophage migration inhibitory factor (MIF)—a pleiotropic cytokine with proinflammatory properties—is also elevated in SSc-PAH patients (Stefanantoni et al., 2015). The level of IL-5, IL-8, and IL-12 show no difference between SSc patients with and without PH (McMahan et al., 2015). Bosentan, a drug used for PAH therapy, causes a significant decrease of the serum levels of IL-2, IL-6, IL-8, and IFN-γ (Bellisai et al., 2011).

The growth differentiation factor (GDF)-15—a member of the transforming growth factor (TGF)-β superfamily- is strongly elevated in remodeled pulmonary arteries of SSc-PAH patients (Nickel et al., 2011). Accordingly, serum levels are increased in SSc-PAH patients compared with SSc patients without PAH and positively correlate with SPAP (Meadows et al., 2011). Importantly, an increased level of GDF-15 is associated with increased mortality (Nickel et al., 2008).

The level of acute phase response protein pentraxin-3 (PTX3), which act as an antiangiogenic factor by binding to fibroblast growth factor-2 (FGF-2) and inhibiting FGF-2-dependent neovascularization and extracellular matrix (ECM) proliferation is increased, while the level of fibroblast growth factor-2 (FGF-2) is decreased in SSc-PAH (Rusnati et al., 2004). Moreover, both changes are independently associated with the presence of PAH (Shirai et al., 2015). Markers associated with inflammation and immune activation are indicated in Table 2.

**Table 2:** Markers of inflammation and immune activation in SSc-PAH patients.

**Reference**	**Marker**	**No. SSc patients with PAH**	**No. SSc patients without PAH**	**No. control subjects**	***P*-value**
George et al., 2014	IFN	28	35	9	n.s.^*^
	IP-10				<0.05^*,**^
	ET-1				<0.05^*,**^
	IL-6				<0.05^**^
	IL-12p70				<0.05^*^
	TNF-α				<0.05^**^
van Bon et al., 2014	CXCL-4	n.d.	n.d.	n.d.	<0.001^**^
Christmann et al., 2011	IL-13	13	22	10	<0.001^*^
McMahan et al., 2015	IL-5	37	40	–	n.s.
	IL-8				n.s.
	IL-12				n.s.
Meadows et al., 2011	GDF-15	30	24	13	=0.004^**^
Gialafos et al., 2008	TIMP-4	37^#^	69	–	=0.003
Shirai et al., 2015	PTX3	21	150	–	=0.006
Lorenzen et al., 2010	OPN	8	62	–	=0.001

*ET-1, endothelin 1; GDF-15, growth differentiation factor-15; IFN, interferon; IL, interleukin; IP-10, interferon gamma (symbol) inducible protein 10; n.d., no data available; n.s., no significant; OPN, osteopontin; PTX3, pentraxin 3; TIMP, tissue inhibitor of matrix metalloproteinase-4; TNF-alpha(symbol), tumor necrosis factor alpha(symbol)*.

* *SSc-PAH vs. Control*.

** *SSc vs. SSc-PAH*.

# *SSc patients with elevated pulmonary artery systolic pressure (>=40 mmHg)*.

## Extracellular matrix components

Matrix metalloproteinases (MMPs) together with their inhibitors (TIMPs) are responsible for the degradation of ECM proteins and lead to the release and activation of cytokines, growth factors but also ECM degradation products (Nagase et al., 2006).

Osteopontin (OPN) is an extracellular matrix protein involved in bone remodeling, but it is also involved in pro-inflammatory and pro-fibrotic properties via modulation of a variety of cell types, including endothelial and vascular smooth muscle cells (Anborgh et al., 2011). It is elevated in SSc patients with PH, however, the same is true in SSc patients with ILD (Lorenzen et al., 2010). Unfortunately, there are no data comparing OPN serum or plasma levels between SSc-PAH and SSc-ILD patients. OPN has also been associated with IPAH (Lorenzen et al., 2011) and is therefore not specific for SSc-PAH. Circulating pro-MMP-10 was increased, in SSc-PH patients in comparison with SSc patients without PH or controls, which is consistent with MMP-10 overexpression in the pulmonary arteries of SSc-PAH patients (Avouac et al., 2017). The matrix metalloproteinase tissue inhibitor-4 (TIMP-4) may contribute to extracellular matrix deposition in SSc and its level is correlated with elevated SPAP in SSc patients (Gialafos et al., 2008). However, such correlations have also been found in PAH patients without SSc (Tiede et al., 2016). MMP-12 was elevated in capillary vessels of SSc-ILD patients, while MMP-7 in blood of SSc-ILD patients. However, the blood level of them were not analyzed in SSc-PAH patients (Moinzadeh et al., 2011; Manetti et al., 2012).

MMPs seem to be increased in SSc-ILD patients, while TIMPs are more likely associated with vascular changes. However, there are no prospective studies comparing these molecules between SSc-ILD and SSc-PAH patients.

## Endothelial physiology and angiogenesis

Microvascular endothelial cell injury plays a pivotal role in the pathogenesis of SSc (Altorok et al., 2014). The disease is characterized by an elevated number of activated monocytes/macrophages or T-lymphocytes in the circulation and tissues (Hasegawa et al., 2014). The infiltration of internal organs by these cells may provoke endothelial damage, fibroblast abnormalities, and alternatively activated macrophages, through the release of a variety of chemokines, cytokines, or growth factors (Abraham et al., 2009; Ueda-Hayakawa et al., 2013). Clinical and pathological findings of vascular destruction and endothelial cell activation strongly support the hypothesis of a unique vascular disease accompanied by the presence of inflammatory and redox potential changes (Abraham and Distler, 2007). Several soluble markers associated with endothelial damage, including a wide spectrum of adhesion molecules, anti-endothelial antibodies, or endothelial progenitor cells are increased in the circulation of SSc-PAH patients and thus may serve as potential biomarkers of a pulmonary vascular involvement.

### Endothelin 1

Cell adhesion molecules located on the surface of endothelial cells are involved in cell adhesion and endothelial cell-smooth muscle cell interactions. Endothelin 1 (ET-1) is a potent vasoconstrictor peptide that is mainly secreted from endothelial cells (Hickey et al., 1985). This mechanism is triggered by protein kinase C (PKC) activation via enhancing the production of 1,2-diacylglycerol in vascular muscle cells (Barman, 2007). However, in pathological conditions ET-1 is secreted by many other cells, including fibroblasts, epithelial cells, smooth muscle cells, or inflammatory cells, such as macrophages and leukocytes (Böhm and Pernow, 2007). In fibroblasts, the expression of the peptide is induced by TGF-β causing also fibroblast migration, myofibroblast differentiation and proliferation of smooth muscle cells. Endothelin exerts its biological activity by interacting with two cell membrane-bound receptors called ET receptor A (ET_A_R) and B (ET_B_R). ET receptor antagonists are approved as targeted medications for PAH and one of them, bosentan, is also approved for the prevention of new digital ulcers in SSc patients (Hamaguchi et al., 2017).

An early study showed that ET-1 is elevated in the plasma of SSc patients (Yamane et al., 1991). Additionally, its level is increased in SSc patients with PAH (Coral-Alvarado et al., 2009; Kim et al., 2010) and correlates with echocardiographic parameters of right ventricular (RV) overload (Ciurzynski et al., 2014). According to a prospective observational study, the peptide level could reflect the presence and severity of PH and may indicate the response to bosentan therapy in patients with SSc-PH (Kawashiri et al., 2014). However, circulating ET-1 levels depend very much on ET-1 clearance by ET_B_R on endothelial cells and may not represent the ET-1 levels in the tissues of interest.

### Circulating endothelial cells and endothelial progenitor cells

Circulating endothelial cells (CECs) and endothelial progenitor cells (EPCs) may play a role in endothelial repair and neovascularization and serve as biomarkers of PAH (Foris et al., 2016). Moreover, there is an evidence of dysfunction of these cells in PAH (Toshner et al., 2009). Regarding CECs in SSc, they were significantly correlated with PAP and DLCO in lcSSc patients (Del Papa et al., 2004). Previous studies suggested that EPC-derived endothelial cells (ECs) may play a role in the progression of vascular complications in SSc (Avouac et al., 2008a,b). A reduced number of EPCs was associated with PAH in SSc (Nevskaya et al., 2008). EPC-derived ECs showed an upregulation of the matrix metalloproteinase-10 (MMP-10) gene in SSc-PAH.

### Asymmetric dimethylarginine

Asymmetric dimethylarginine (ADMA) is an endogenous inhibitor of eNOS, which may contribute to endothelial dysfunction. In a small cohort of SSc-PAH patients, ADMA levels were significantly associated with PAH after adjustment for specific disease characteristics, cardiovascular risk factors, and other related vascular complications (Thakkar et al., 2016). An ADMA level ≥0.7 ng/mL in combination with an NT-proBNP ≥210 ng/mL showed 100% sensitivity and 90% specificity for the identification of SSc-PAH (Thakkar et al., 2016). However, other studies did not find any significant correlations between ADMA and echocardiographic markers of PH, (Dag et al., 2014; Foris et al., 2016), although they found a negative correlation with the 6-minute walking test (6MWT) (Dimitroulas et al., 2008). Taken together, the role of ADMA as a biomarker is currently controversial.

### Von willebrand factor, vascular endothelial growth factor, endostatin

Von Willebrand Factor (vWF) is a circulating glycoprotein and a marker of endothelial cell activation or damage, secreted by endothelial cells and megakaryocytes. It plays an important role in the coagulation cascade as a carrier for coagulation factor VIII (Lip and Blann, 1997). Elevated levels of vWF were found in IPAH and in CTEPH patients (Bonderman et al., 2003) and also in lcSSc patients with PAH which was associated with an increased risk for a PAP elevation (Pendergrass et al., 2010; Barnes et al., 2012). In contrast, in another study, there was no difference between SSc and SSc-PAH patients in vWF levels (Iannone et al., 2008). In addition, the vWF antigen was elevated in MCTD-PAH patients, as compared to MCTD patients without PAH (Vegh et al., 2006). Thus, vWF may be a marker of increased PAH risk in lcSSc and MCTD patients. No data, however, are available for dcSSc patients.

The angiogenic factor vascular endothelial growth factor (VEGF) is increased in SSc patients with elevated SPAP as assessed by echocardiography. Additionally, there is a positive correlation between VEGF and SPAP (Papaioannou et al., 2009).

Endostatin is a potent angiostatic peptide, which is a cleavage product of the extracellular matrix protein, collagen 18. Indeed, it may be considered as an endogenous antagonist of VEGF. It is massively upregulated in the intima of remodeled pulmonary arteries from SSc-PAH patients, and circulating levels of endostatin are correlated with markers of right ventricular failure (Hoffmann et al., 2015). Endostatin levels were elevated in SSc patients as compared to control subjects and a multivariable analysis in SSc patients showed an association between elevated endostatin levels and PAH. Endostatin was also a strong predictor of mortality (Reiseter et al., 2015). There is a polymorphism of collagen 18a1 which alters circulating endostatin levels and is also strongly associated with mortality in SSc patients. Finally, endostatin serum levels are correlated with exercise capacity, World Health Organization (WHO) functional class, and pulmonary hemodynamics (Damico et al., 2015).

### Other endothelial markers

Other factors associated with endothelial physiology, such as platelet endothelial cellular adhesion molecule-1 (PECAM-1) and endoglin were also investigated in SSc-PAH. Increased PECAM-1 was found in SSc patients with digital ulceration and PAH, however, correlations with clinical parameters were not significant (Riccieri et al., 2011). In SSc-PAH compared to healthy controls, the endoglin level was increased and correlated with circulating ET-1 levels (Coral-Alvarado et al., 2009). However, the diagnostic and predictive value of these markers has not been confirmed in prospective studies. The soluble forms of E-selectin (sE-selectin) and vascular cell adhesion molecule-1 (sVCAM-1) serum levels were not elevated in lcSSc patients (Stratton et al., 1998). In accordance, another study found also no difference between the sVCAM levels of SSc and SSc-PAH patients (Iannone et al., 2008). Endothelial cells not only secrete various mediators but they can also release exosomes, a cell-derived vesicles. Exosomes can contain various macromolecules including proteins, lipids, and nucleic acids such as microRNA. Therefore, either their content or exosomes *per se* can serve as biomarkers. They may play a role in extension of fibrotic SSc process in non-affected tissues (Wermuth et al., 2017). The blood level of exosomes in SSc patients with vascular involvements were decreased (Nakamura et al., 2016b). However, further studies are required to prove their role in the vascular pathological processes of SSc-PAH patients.

## Metabolic changes

There is strong experimental and epidemiological evidence supporting a “metabolic theory” of PAH development. Accordingly, several organs of PAH patients share mitochondria-based metabolic changes (Paulin and Michelakis, 2014; Michelakis et al., 2017).

### Adipocytokines

In SSc-PAH, dysregulated adipose tissue and adipokine dysbalance have been found. The adipocytokines such as resistin, leptin, adiponectine, adipsin, or omentin are soluble and circulating factors. They are mainly produced by adipocytes and have pro-inflammatory and pro-angiogenic properties (Tilg and Moschen, 2006). Leptin has been considered as a mediator of immunological disorders in IPAH. Its level was elevated in IPAH and SSc-PAH patients compared to healthy controls and the function of leptin expressing T-lymphocytes was impaired in a leptin-dependent manner. However, leptin levels were not different between IPAH and SSc-PAH patients (Huertas et al., 2012). Omentin was also elevated in SSc patients with increased SPAP, however, it was not correlated with any fibrotic or inflammatory parameters (Miura et al., 2015). In SSc patients, SPAP was also associated with elevated resistin levels (Masui et al., 2014). Elevated circulating levels of adipsin were associated with SSc-PAH and adipsin gene single-nucleotide polymorphisms (Korman et al., 2017).

### 25(OH)-D vitamin

In patients with SSc, low serum 25(OH)-D Vitamin levels were associated with increased SPAP as assessed by echocardiography (Atteritano et al., 2016) and there was a significant correlation between serum levels and diastolic dysfunction (Groseanu et al., 2016), but not with pulmonary arterial pressure (Groseanu et al., 2016). However, there are few diseases that have not been associated with decreased 25(OH)-D vitamin levels. Therefore, this is certainly not specific for SSc or for PAH.

### Metabolomics

In recent years, metabolomics showed promising results in the field of pulmonary vascular research. In an exploratory approach, numerous metabolites were associated with pulmonary arterial pressure and the elevation of kynurenine appeared quite specific for PH (Lewis et al., 2016). Indeed, kynurenine is a strong endogenous pulmonary vasodilator increasing both cAMP and cGMP levels in the target cells (Nagy et al., 2017). This suggests that the kynurenine system represents a negative feedback mechanism for PH, similar to the natriuretic peptides. In addition, the kynurenine system has a strong impact on immunologic signaling (Jasiewicz et al., 2016). Moreover, a recent analysis based on orthogonal signal correction (OSC), combined with a method of two dimensional separation of NMR data, highlighting possible clusters, trends, or outliers, confirmed a change in the metabolic profile of SSc-PAH as compared to SSc without PAH (Deidda et al., 2017). Altogether this suggests that many metabolic factors are changed in PAH, however, it is not clear if they are cause or consequence of the disease and what is their role in the pathogenesis of SSc-PAH.

## MicroRNAs

Epigenetic changes are heritable alterations of the human genome affecting the gene expression without involving changes of the underlying DNA sequences. As the pathogenesis of SSc is thought to be influenced by environmental factors affecting human genome, these stimuli have been considered to be responsible for epigenetic regulatory complex changes which can manifest in alterations in disease outcomes (Aslani et al., 2018). RNA interference via microRNAs is considered to be one of the potential mechanisms to initiate and maintain epigenetic changes. Alterations in the regulation of microRNAs may lead to pathway alterations playing a role in the development of PAH (Thenappan et al., 2018). Moreover, they might contribute in processes of right ventricular remodeling (Batkai et al., 2017). According to these concepts, microRNAs can be identified in the circulation, and circulating miRNA levels vary according to the severity of PH (Wei et al., 2013; Zhao et al., 2017). In patients with PH, the level of circulating miR-424(322) was elevated and was associated with more severe symptoms and hemodynamic changes, while miR-4632 has been identified as a possible serum PAH biomarker (Qian et al., 2017; Baptista et al., 2018). Regarding SSc miR-193b, it has been described as a possible contributor to proliferative vasculopathy (Iwamoto et al., 2016). In addition, microRNA let-7d from skin biopsies showed a negative association with the severity of PAP measured by echocardiography in patients with SSc (Izumiya et al., 2015). In summary, based on results in PAH and SSc patients, micro RNAs might represent attractive biomarkers as well as future therapeutic targets in PH and SSc. However, their role in the pathogenesis of SSc-PAH needs further investigation.

## Markers of cardiac dysfunction

Microvascular alterations may play a pivotal role both in the impairment of myocardial function and the development of pulmonary vascular disease in SSc. These changes, directly or indirectly may cause right ventricular failure. Several studies investigated the potential role of different markers released by the heart, including the natriuretic peptide family, D-dimer as well as Troponin T and I as diagnostic and prognostic tools for PH in SSc patients. Studies investigating heart-related markers in SSc associated PH are listed in Table 3.

**Table 3:** Overview of heart related markers in patients with SSc-PAH or at risk of PH, correlation with hemodynamic parameters, predictive value, cut-off values, and association with survival.

**Reference**	**Marker**	**No. SSc-PAH patients**	**RVSP**	**mPAP**	**PVR**	**Independent predictive ability for PAH (Odds ratio)**	**Cut-off value for identification of PAH**	**Association with survival**
Költo et al., 2014	NT-proANP	144^*^	–	–	–	–	822.5 pmol/l (Sensitivity: 56.3% Specificity: 79.5%)	yes
	NT-proBNP						154.5 pmol/l (Sensitivity: 50% Specificity: 76.8%)	
Mukerjee et al., 2003	NT-proBNP	23	*r* = 0.59	*r* = 0.53	*r* = 0.49	–	395.34 pg/ml (Sensitivity:0.69 Specificity: 1.0)	–
Ciurzynski et al., 2008	NT-proBNP	51^*^	–	–	–	29.5	115 pg/ml (Sensitivity: 92% Specificity: 44%)	–
Cavagna et al., 2010	NT-proBNP	20	–	*r* = 0.61	*r* = 0.61	–	239.4 pg/ml (Sensitivity: 45% Specificity: 90%)	–
	BNP		–	*r* = 0.72	*r* = 0.61	2.1	64 pg/ml (Sensitivity: 60% Specificity: 87%)	–
Thakkar et al., 2016	NT-proBNP	15 (all 94)	*r* = 0.65^**^	*r* = 0.63^**^	*r* = 0.76^**^	–	209.8 pg/ml (Sensitivity: 92.9% Specificity: 100%)	–
Allanore et al., 2008	NT-proBNP	8	–	–	–	6.35 (*p* = 0.053)	–	–
Williams et al., 2006	NT-proBNP	68	–	*r* = 0.62	*r* = 0.81	–	91 pg/ml (Sensitivity: 90% Specificity: 51%)	yes
Rotondo et al., 2017	NT-proBNP	21	*r* = 0.30	–	–	–	–	–
Kiatchoosakun et al., 2007	D-dimer	47	n.s.	–	–	–	–	–
Nordin et al., 2017	NT-proBNP	44^#^	–	–	–	1.9	–	–
	Hs-cTnI		–	–	–	3.2	–	–
Avouac et al., 2015a	NT-proBNP	89^&^	–	–	–	26.6	–	–
	Hs-cTnT		–	–	–	2.0	–	–
	NT-proBNP + Hs-cTnT		–	–	–	50.0	–	–

*BNP, brain natriuretic peptide; Hs-cTnI, high-sensitivity cardiac troponin I; Hs-cTnI, high-sensitivity cardiac troponin T; NT-proANP, N-terminal atrial natriuretic peptide; NT-proBNP, N-terminal pro brain natriuretic peptide. All values reached the significance level of p < 0.05*.

* *SSc patients with heart involvement, including PH*.

** *The analysis involved all the patients*.

# *SSc patients with abnormal echocardiographic findings*.

& *SSc patients with cardiovascular risk factors*.

### Natriuretic peptides

Natriuretic peptides are well established, clinically useful markers of right ventricular dysfunction in PH. A-type natriuretic peptide (ANP) is secreted from granula in the atrial cardiomyocytes in response to an increased RV afterload. Any release of afterload causes an immediate decrease of the secretion (Wiedemann et al., 2001). The major disadvantage of ANP lies in its complicated handling methods. The N-terminal fragment of A-type natriuretic peptide (NT-proANP) is the inactive form of ANP, which is more stable and has a longer life-time in the circulation. In a prospective study, NT-proANP revealed a prognostic value for cardiac involvement, including PH in SSc (Költo et al., 2014).

Most of the studies focused on the investigation of BNP and its terminal fragment NT-proBNP. NT-proBNP is a 32-amino acid polypeptide attached to a 76-amino acid N-terminal fragment and it is secreted but not stored by ventricular cardiomyocytes (Janda and Swiston, 2010). BNP does not need cooled handling or transportation after blood drawn and the metabolic clearance of NT-proBNP is slow in comparison with ANP or BNP (Foris et al., 2013). As a consequence, the levels depend considerably on renal function.

Several studies found significant correlations between hemodynamic parameters, exercise capacity and natriuretic peptides in SSc-PAH (Mukerjee et al., 2003; Ciurzynski et al., 2008; Dimitroulas et al., 2010). In screening for PAH, both BNP and NT-proBNP were correlated with PAP, and BNP was an independent predictor of PAH in SSc patients (Cavagna et al., 2010). NT-proBNP combined with pulmonary function test and other markers had a high sensitivity and specificity in a screening model for PH (Thakkar et al., 2016). Importantly, NT-proBNP has been included in the 2015 risk stratification for IPAH as a prognostic marker (Galiè et al., 2015). A study in 101 SSc patients found that an increased NT-proBNP level together with a decreased DLCO/VA ratio was highly predictive for PAH development in the next 29-months (Allanore et al., 2008). Moreover, in a prospective study, the peptide level alone was strongly related to the severity of PAH and its increase during therapy was associated with high mortality (Williams et al., 2006). Another study found no relation between the changes of NT-proBNP and the clinical status (Rotondo et al., 2017). A retrospective study in 432 SSc patients with PH due to left heart disease from a French-Canadian cohort suggested mid-regional pro-atrial natriuritic peptide (MR-proANP) and mid-regional pro-adrenomedullin (MR-proADM) may be more reliable than NT-proBNP as a biomarker for early PH (Miller et al., 2014).

### D-dimer, troponin T and I

There is some indication from epidemiological and experimental studies that microvascular thrombosis may be involved in the pathogenesis of PAH. However, a cross-sectional study in SSc-PAH patients found no correlation between plasma D-dimer and RVSP assessed by echocardiography (Kiatchoosakun et al., 2007).

Troponin T (TnT) and high-sensitive Troponin I (hs-cTnI) are well-known markers of acute ischemic heart disease and have been identified as independent markers of mortality in PAH (Foris et al., 2013). A small study in SSc patients found a significant association between hs-cTnI and elevated echocardiographic SPAP (Nordin et al., 2017). The high-sensitive TnT (HS-cTnT) was even elevated in SSc patients without relevant cardiovascular risk factors and an HS-cTnT level of >14 ng/L was independently associated with PAH. The combination of this marker with NT-proBNP was strongly associated with PAH (Avouac et al., 2015a). Therefore, the combination of TnT subtypes and NT-proBNP might serve as predictor for PH in SSc. Unfortunately, these markers are not specific for the right ventricle and may be increased to the same extent by left heart disease. They are also not specific for SSc or any other cause of PH.

## Future clinical and research needs

The diagnosis of SSc-PAH needs an invasive method, therefore the inauguration of a well-established non-invasive diagnostic method would be crucial. The number of studies evaluating biomarkers in blood samples as diagnostic tools for PAH detection in SSc is progressively increasing, however very few of them have demonstrated solid diagnostic performance. Recent advances in the understanding of pathophysiological processes are promising for further therapies; nevertheless, the most important point for now is the early diagnosis as a mean to early treatment. The combination of biomarkers which help to differentiate between pulmonary parenchymal and vascular complications in SSc at an early stage would be very important. However, these biomarkers have to be validated in prospective multicenter studies involving a large series of patients. In addition, there are no unified definitions to segregate PAH (group 1 of the World Classification of PH) from PH-ILD (group 3 of the World Classification of PH). Different studies apply different definitions that make it difficult to compare the data about potential biomarkers. Thus, selection criteria for patients must be defined well and prospectively. Finally, an extended research interest is needed implicating underlying mechanisms described in systemic sclerosis. One example may be the association between adipocytokines and malabsorbtion, as latter molecules can be associated with the disease pathogenesis. It is likely that in the future some of the discussed biomarkers will be employed, alone or in combination with other already established biomarkers or clinical parameters, to improve the accuracy of early diagnosis and guide therapy.

## Conclusions

PAH is a severe complication of SSc and associated with high morbidity and mortality. There are several biomarkers of SSc-PAH, reflecting endothelial physiology, inflammation, immune activation, extracellular matrix, metabolic changes, or cardiac involvement. Biomarkers in form of antibodies, cytokines, chemokines, metabolites, and natriuretic peptides were associated with diagnosis, disease severity, and progression. However, very few have been tested in a prospective setting. Prospective studies in well-defined patient cohorts are warranted to develop reliable algorithms for detection and prognosis of SSc-PAH.

## Author contributions

All authors conceptualized and designed the review. BO, HO, and GK wrote the paper. VF, AG, VM, GKW and PH provided critical feedback and input. All authors agree to be accountable for the content of the work and approved the manuscript.

### Conflict of interest statement

GK reports personal fees and non-financial support from Actelion, personal fees and non-financial support from Bayer, personal fees and non-financial support from GSK, personal fees and non-financial support from MSD, personal fees and non-financial support from Boehringer Ingelheim, personal fees and non-financial support from Novartis, personal fees and non-financial support from Chiesi, non-financial support from VitalAire, outside the submitted work. HO reports grants, personal fees and non-financial support from Actelion, grants, personal fees and non-financial support from Bayer, personal fees and non-financial support from GSK, personal fees from Novartis, personal fees from Astra Zeneca, grants, personal fees and non-financial support from Boehringer, personal fees and non-financial support from Chiesi, personal fees and non-financial support from Menarini, grants and personal fees from Roche, personal fees from Bellerophon, personal fees and non-financial support from TEVA, personal fees and non-financial support from MSD, personal fees and non-financial support from Ludwig Boltzmann Institute for Lung Vascular Research, outside the submitted work. The remaining authors declare that the research was conducted in the absence of any commercial or financial relationships that could be construed as a potential conflict of interest.
